# The Biocontrol Agent *Pyemotes zhonghuajia* Has the Highest Lethal Weight Ratio Compared with Its Prey and the Most Dramatic Body Weight Change during Pregnancy

**DOI:** 10.3390/insects12060490

**Published:** 2021-05-25

**Authors:** Yi-Chai Chen, Tai-An Tian, Yi-Hui Chen, Li-Chen Yu, Ji-Feng Hu, Xiao-Fei Yu, Jian-Feng Liu, Mao-Fa Yang

**Affiliations:** 1 Guizhou Provincial Key Laboratory for Agricultural Pest Management of the Mountainous Region, Institute of Entomology, Scientific Observing and Experimental Station of Crop Pest in Guiyang, Ministry of Agriculture, Guizhou University, Guiyang 550025, China; yichaichen@126.com (Y.-C.C.); taian2015@126.com (T.-A.T.); 2 Guizhou Institute for Metrology and Calibration, Guiyang 550003, China; 18798012759@163.com; 3 Changli Institute of Pomology, Hebei Academy of Agriculture and Forestry Sciences, Changli 066600, China; ylc825@hotmail.com; 4 Journal Editorial Department, Guizhou University, Guiyang 550025, China; jifenghu28@163.com; 5 College of Tobacco Science, Guizhou University, Guiyang 550025, China; xfyu1@gzu.edu.cn

**Keywords:** *Pyemotes zhonghuajia*, venom, parasite *Spodoptera litura*, biocontrol, opisthosoma

## Abstract

**Simple Summary:**

To explore the biocontrol potential of *Pyemotes zhonghuajia*, we studied the paralytic process, time to lethality, efficiency, reproductive development, and the ability to search for *Spodoptera litura* larvae. We found that one newly emerging *P. zhonghuajia* female could kill a third instar larva greater than 680,000 times her own body weight. An individual *P. zhonghuajia* adult female was able to kill *S. litura* eggs and first to third instar larvae. *Pyemotes zhonghuajia* could develop on eggs, first to sixth instar larvae, and pupae, but only produced offspring on the eggs and pupae. The presence of 50 or 100 *P. zhonghuajia* resulted in significantly higher mortality rates of first, second, and third instar *S. litura* larvae in a 2 cm searching range compared with other searching ranges (4.5 and 7.5 cm). Therefore, *P. zhonghuajia* could potentially be used to control *S. litura*.

**Abstract:**

*Pyemotes* spp. are small, toxic, ectoparasitic mites that suppress Coleoptera, Hemiptera, and Lepidoptera plant pests. To explore their potential use as a biocontrol agent, we studied the reproductive development, paralytic process, time to lethality and mortality, and searching ability of *Pyemotes zhonghuajia* on different developmental stages of the oriental leafworm moth, *Spodoptera litura*. *Pyemotes zhonghuajia* gained 14,826 times its body weight during pregnancy. One single *P. zhonghuajia* female could rapidly kill one *S. litura* egg and first to third instar larvae, but not fourth to sixth instar larvae, prepupae, or pupae within 720 min. *Pyemotes zhonghuajia* could develop on eggs, first to sixth larvae, and pupae, but only produced offspring on the eggs and pupae. A single *P. zhonghuajia* female (an average weight of 23.81 ng) could paralyze and kill one *S. litura* third instar larvae (an average weight of 16.29 mg)—680,000 times its own weight. Mites significantly affected the hatch rate of *S. litura* eggs, which reduced with increasing mite densities on *S. litura* eggs. Releasing 50 or 100 *P. zhonghuajia* in a 2 cm searching range resulted in significantly higher mortality rates of *S. litura* first instar larvae within 48 h compared to second and third instar larvae in searching ranges of 4.5 and 7.5 cm within 24 h. To the best of our knowledge, this is the first study to reveal that *P. zhonghuajia* undergoes the greatest changes in weight during pregnancy of any adult female animal and has the highest lethal weight ratio of any biocontrol agent.

## 1. Introduction

*Pyemotes zhonghuajia* Yu, Zhang, and He (Prostigmata: Pyemotidae) is a newly discovered, dominant ectoparasitic mite native to China [1]. This mite is an efficient parasite against forest and agricultural pests, such as *Monochamus alternatus* Hope, *Anoplophora glabripennis* (Motschulsky), *Semanotus bifasciatus* (Motschulsky), *Diaphorina citri* Kuwayama, *Spodoptera frugiperda* (Smith), and *Mythimna separata* (Walker) [2,3,4,5,6,7]. *Pyemotes zhonghuajia* have a lethal effect on the first to fourth instar larvae of *M. alternatus* [2]. Releasing 2000 *P. zhonghuajia* per orange jasmine plant reduced the population of *D. citri* adults by 89.67% within ten days in the field [5]. This mite is viviparous, and the eggs develop into adult mites and are laid out from the mother’s opisthosoma when they are mature [8]. The opisthosoma size of *P. zhonghuajia* is normally between 558 and 960 μm, and host stages of *M. separata* can influence the increment of opisthosoma size and fecundity of *P. zhonghuajia* [7]. *Pyemotes zhonghuajia* develops and reproduces better on *M. separata* eggs, prepupae, and pupae compared with larvae [7]. Third instar larvae of *M. alternatus* are a suitable host for the development of *P. zhonghuajia* compared with second and fourth instar larvae [2]. However, little is currently known about the weight dynamics of *P. zhonghuajia*.

*Pyemotes* spp. are, in general, venomous ectoparasitic mites, especially considering the high prey to parasite weight ratio: one *Pyemotes tritici* (LaGreze-Fossat and Montagne) female, weighing approximately 300 ng, can permanently paralyze individual *Reticulitermes* spp. larva weighing 6–7 mg within 30 min [9]. *Pyemotes* spp. venom is extremely potent and able to disrupt neuromuscular function in various species of host and non-host organisms, including those belonging to the orders Lepidoptera, Hemiptera, and Coleoptera [9,10]. The venom contains both high molecular weight (TxP-HMW) and low molecular weight (TxP-LMW) fractions, as demonstrated in whole venom extracts from *P. tritici*, which induce flaccid paralysis and paralysis involving immediate muscular contractions, respectively [9]. The extremely potent venom of *Pyemotes zivocljeri* (Krczal) is secreted from the paired glands in the basal part of the pedipalps [10]. Based on the toxin gene sequences of *P. tritici*, twelve sequences were cloned from *P. zhonghuajia,* and the amino acid sequence similarity with the toxin gene sequences of *P. tritici* was 84.21–88.81% [11]. When the crude toxin extract of *P. zhonghuajia* was injected into the fifth instar larvae of *Helicoverpa armigera* (Hübner), the larvae were paralyzed after 30 min, and the virulence effect was significant [12]. In previous research, we found that *P. zhonghuajia* is a potential biocontrol agent against the fall armyworm *S. frugiperda* and northern armyworm *M. separata* [6,7], which are pests of agriculturally important crops. *Pyemotes zhonghuajia* is able to efficiently parasitize their eggs, larvae, and pupae; one *P. zhonghuajia* female has the potential to kill over 50% of *S. frugiperda* and *M. separata* first to third instar larvae within 72 h under laboratory conditions [6,7]. However, there is currently little information on the parasitic and developmental processes of *P. zhonghuajia*, despite its potential as a biocontrol agent against agriculturally significant pests.

This study therefore aimed to characterize the reproductive development, paralytic process, mortality, and searching ability of *Pyemotes zhonghuajia* on different developmental stages of the oriental leafworm moth *Spodoptera litura* (Fabricius) (Lepidoptera: Noctuidae). This species is a widely distributed, polyphagous insect pest that attacks more than 300 species of host plants in subtropical and tropical regions [13]; thus, a natural biocontrol agent against this species such as *P. zhonghuajia* would be valuable, making it a suitable host choice for our study.

## 2. Materials and Methods

### 2.1. Insect Cultures for Bioassays

The *Spodoptera litura* used in this study were initially collected in August 2020 from Fenggang county, Guizhou Province, China. Larvae were brought back to the laboratory and reared on leaves of K326 tobacco plants for 3 generations in a climate chamber before the experiment with a temperature of 25 ± 2 °C, 70 ± 5% relative humidity, and a photoperiod of 10:14 h (light:dark). To obtain populations of *S. litura* larvae of the same age, we randomly placed each pair of mature *S. litura* into a 1000 mL plastic cup containing a cotton ball soaked with 10% honey solution as food. Newly laid eggs (<24 h) were transferred to a 6.5 × 4 × 5.5 cm plastic box containing fresh K326 tobacco leaves. Eggs, larvae, prepupae, and pupae of *S. litura* were then prepared for use in the experiments.

*Pyemotes zhonghuajia* were initially obtained from the Hebei Academy of Agriculture and Fruit Sciences and reared on *M. separata* pupae following the approach described by Tian et al. [7].

### 2.2. Development of P. zhonghuajia on S. litura

To measure the opisthosoma (abdomen) of *P. zhonghuajia* on *S. litura* at different life stages, we first transferred 40 mites to single first to sixth instar stage and pupae of *S. litura*. *Spodoptera litura* were left for 24 h, and all but one mite was removed from the surface of each *S. litura* to observe the opisthosoma of *P. zhonghuajia* under a digital video microscope until the death of females. We also recorded the survival time of each female on different stages of *S. litura*. Each treatment was replicated twenty times.

The daily growth of the opisthosoma (*y*) was calculated according to the following Equation (1):

*y* = *a*(*1*−*e^−bx^*) (1)

where *a* is the maximum opisthosoma size of *P. zhonghuajia*, *b* is the estimated rate of increase in opisthosoma size [7,14,15], and *x* is the number of days after the removal of all but one mite.

The weights of *P. zhonghuajia* and *S. litura* were measured using a comparator balance (Mettler XP56) with 1 μg (0.001 mg) sensitivity. As newly emerging female mites (<12 h) were very small, we weighed a total of 170 mites per replicate. The following equations (2–4) were used to calculate the weight of 170 mites (*∆m*):

*m*1 = *B* − (*A*1 + *A*2)/2 + 0.008 mg + 500 mg (2)

*m*2 = *B* − (*A*1 + *A*2)/2 + 0.008 mg + 500 mg (3)

*∆m* = *m*2 − *m*1 (4)

where *m*1 is the weight of the sample pan without adding samples, *m*2 is the weight of 170 mites and the sample pan, *B* is the weight of the sample shown on the balance, *A*1 and *A*2 are the values of two adjacent standard weights on the balance, 500 mg is the mass of each standard weight, and 0.008 mg is the correction value of the 500 mg standard weights.

### 2.3. Parasitic Process and Time to Lethality of P. zhonghuajia to S. litura

One single *P. zhonghuajia* female was transferred to the body of *S. litura* at the first instar larval stage with a fine brush, and the parasitic process of *P. zhonghuajia* was observed under a Keyence VHX-6000 microscope. A JEOL JCM-6000 scanning electron microscope and a Leica DM 3000 microscope were used to observe the mouthpart of *P. zhonghuajia*.

To measure the time to lethality of *P. zhonghuajia* on different *S. litura* at different life stages, we observed the effect of a single female *P. zhonghuajia* mite on the mortality rates of first to sixth instar larvae, prepupae, and pupae of *S. litura* in a 6.5 × 4 × 5.5 cm plastic box for a total duration of 720 min. There were ten replicates for each life stage of *S. litura*. We continually monitored first to third instar larvae until their death and monitored fourth and sixth instar larvae, prepupae, and pupae every 60 min using a high-definition digital video microscope (EVDM20101, Shenzhen Yishijie Optoelectronics Technology Co., Ltd., Shenzhen, China). Ten replicates were conducted for each of the pupal and larval stages.

### 2.4. Mortality of S. litura as a Result of P. zhonghuajia Parasitism

To evaluate the effect of *P. zhonghuajia* on the mortality rate of *S. litura*, we transferred different numbers of adult female *P. zhonghuajia* mites (3, 5, 10, 20, or 40 mites) on either one larva at either the fourth or fifth instar stages of *S. litura* in each experimental arena (a plastic box measuring 6.5 × 4.0 × 5.5 cm) using a fine brush, and counted the number of *S. litura* deaths within 72 h. A controlled experimental arena, which contained no mites, was also set up. There were ten replicates for fourth and fifth instar larvae of *S. litura*. The number of deaths of *S. litura* fourth to fifth instar larvae were counted every 24 h for a total of 72 h.

To assess the influence of *P. zhonghuajia* on the rate of hatching, pupation, and eclosion of *S. litura*, different densities of female *P. zhonghuajia* mites (3, 5, 10, 20, or 40 mites) were transferred onto 100 eggs, one sixth instar larva, one prepupa, or one pupa of *S. litura* in the experimental arena. Numbers of hatching, pupating, and eclosing *S. litura* were also counted daily. There were ten replicates for the eggs of *S. litura* and twenty replicates for the sixth instar larva, prepupa, and pupa.

### 2.5. Searching Ability of P. zhonghuajia on S. litura

To evaluate the searching ability of *P. zhonghuajia* on *S. litura*, we released either 0, 50, or 100 mites into three differently sized experimental arenas [Petri dishes with 4 cm (2 cm searching range), 9 cm (4.5 cm searching range), or 15 cm (7.5 cm searching range) diameter]. Each arena contained the same instar of 20 *S. litura* at the first, second, or third instar stage, and a fresh 4, 9, or 15 cm tobacco leaf disk was placed on moistened cotton in each sized experimental arena. The *P. zhonghuajia* were released in the center of each Petri dish, and *S. litura* were placed along the edge of the dish. We counted the number of deaths of *S. litura* within 48 h. Each treatment was replicated 10 times.

### 2.6. Data Analysis

We used SPSS 26.0 to calculate the mortality rate of *S. litura* at different mite densities and in different searching ranges. The normality of the data was verified using the Kolmogorov–Smirnov test, and homogeneity of variance was tested with the Bartlett test. If the rates of mortality, hatching, pupation, and eclosion did not meet the assumptions of normality or homogeneity, the data were transformed using the arcsine square root transformation. The time to mortality; the rate of mortality, hatching, pupation, and eclosion; longevity; and opisthosoma size of *P. zhonghuajia* under different test conditions were assessed using one-way analysis of variance (ANOVA) and Tukey’s honest significant difference (HSD) multiple range test. Bar graphs for time to mortality and mortality rates were constructed using Origin 2018; the graph for the survival rate of *S. litura* was constructed using GraphPad Prism 8.

## 3. Results

### 3.1. Reproductive Development of P. zhonghuajia on S. litura at Different Life Stages

**Figure 1:**
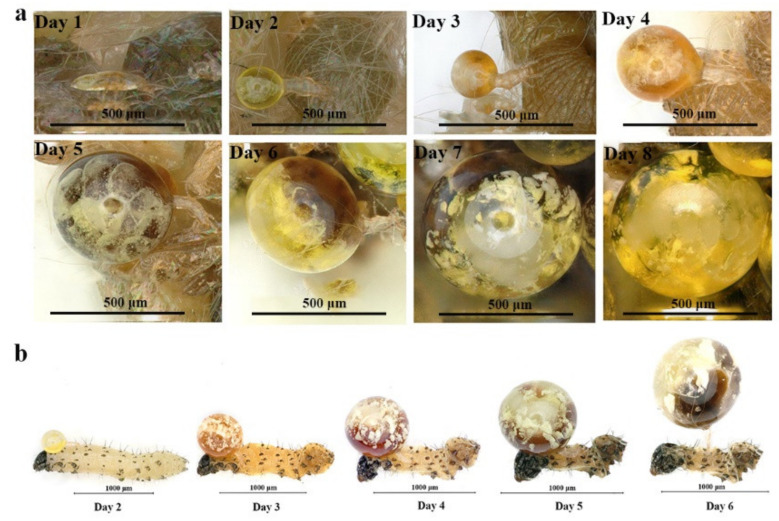
Opisthosoma development in individual *Pyemotes zhonghuajia* females on (**a**) eggs and (**b**) first instar larvae of *Spodoptera litura*.

*Pyemotes zhonghuajia* opisthosoma development on *S. litura* eggs is shown in Figure 1, Figure S1, and Figure S2, and in Table 1. The average weight of single adult *P. zhonghuajia* female (<12 h old) and those in later pregnancy (8 days post-emergence from pupae) on *S. litura* eggs were 23.81 ng and 353,000 ng, respectively. Compared with newly emerging *P. zhonghuajia* females, the weight of *P. zhonghuajia* in later pregnancy was 14,826 times higher. The mouthpart length of *P. zhonghuajia* was 11.3 μm, and the front and base width were 0.376 μm and 0.872 μm, respectively. The location of the paired venom glands was confirmed to be the basal part of the pedipalps of *P. zhonghuajia*, as shown in Figure S3.

The average opisthosoma size of *P. zhonghuajia* on *S. litura* eggs increased from 0.15 mm at day 1 to 0.93 mm on day 8 (Table 1). Opisthosoma color in *P. zhonghuajia* changed along with the color of *S. litura* eggs from grayish yellow, greenish yellow, or light brown, to dark brown and milk white. White flocculent was observed in the opisthosoma of *P. zhonghuajia* on day 4, and the body of the offspring was observed in the opisthosoma on day 8 (Figure 1 and Figure S1).

*Pyemotes zhonghuajia* was able to develop on *S. litura* eggs, first to sixth instar larvae, and pupae, but successfully produced offspring only on eggs and pupae on day 8 (Table 1, Figure S2). *P. zhonghuajia* was unable to survive on first to sixth instar larvae of *S. litura* as the larvae were shriveled over 6 days (Figure 1 and Figure S2). The opisthosoma size of *P. zhonghuajia* on pupae was significantly larger than that of the mites on the eggs of *S. litura*. The maximum opisthosoma size (a = 1156.99 μm, b = 0.28) of *P. zhonghuajia* on pupae were higher than on eggs (a = 1028.44 μm, b = 0.17), but there were no significant differences in longevity and opisthosoma size of *P. zhonghuajia* (Table 1).

### 3.2. The Parasitic Process of P. zhonghuajia on S. litura

When *P. zhonghuajia* mites were transferred to the surface of *S. litura*, the mites bit their hosts after approximately 15 s. *Spodoptera* *litura* could immediately wild crawl, move irregularly, and bite back the bitten position where the mouthparts of *P. zhonghuajia* had punctured the intersegmental cuticle. *Spodoptera litura* first and second instar larvae had decreased activity 8 min and 13 min post-injection, respectively. After approximately 17 min, *S. litura* first and second instar larvae stopped crawling and started twitching, and second instar larvae vomited bodily fluids. After about 15 or 40 min, *S. litura* first or second instar larvae stopped twitching and its body color gradually turned brown; meanwhile, *P. zhonghuajia* began to move to find the best position for feeding and reproduction (Videos S1 and S2).

### 3.3. Time to Lethality of Individual P. zhonghuajia on S. litura

**Figure 2:**
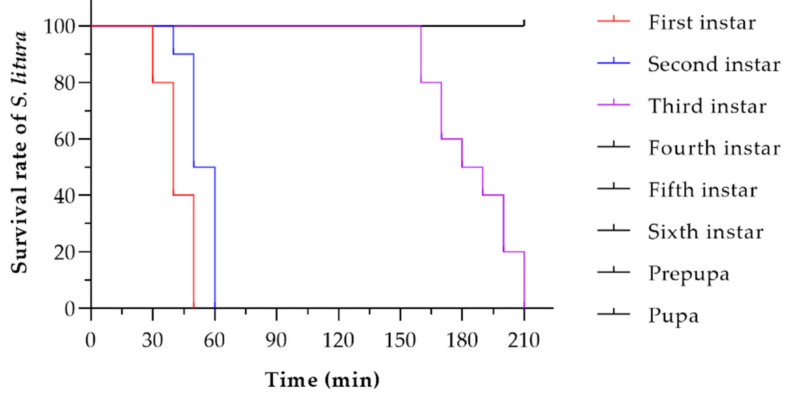
Survival rates of individual first to sixth instar larva, prepupa, and pupa of *Spodoptera litura* parasitized by individual *Pyemotes zhonghuajia* up to 720 min.

An individual *P. zhonghuajia* adult female was able to kill first, second, and third instar larvae, but could not kill fourth to sixth instar larvae, prepupae, and pupae of *S. litura* within 720 min after placement on the surface of their host (Figure 2). One *P. zhonghuajia* was able to kill first and second instar larvae of *S. litura* in 37.1 min and 49.9 min, respectively. However, the time to lethality was significantly longer (179.1 min) on third instar larvae hosts. The average weight of *S. litura* third instar larvae was 16.29 mg. Therefore, we calculated that individual *P. zhonghuajia* females (23.81 ng) were able to paralyze and kill individual third instar *S. litura* larvae that were approximately 680,000 times their own body weight.

### 3.4. S. litura Mortality with Different Population Densities of P. zhonghuajia

**Figure 3:**
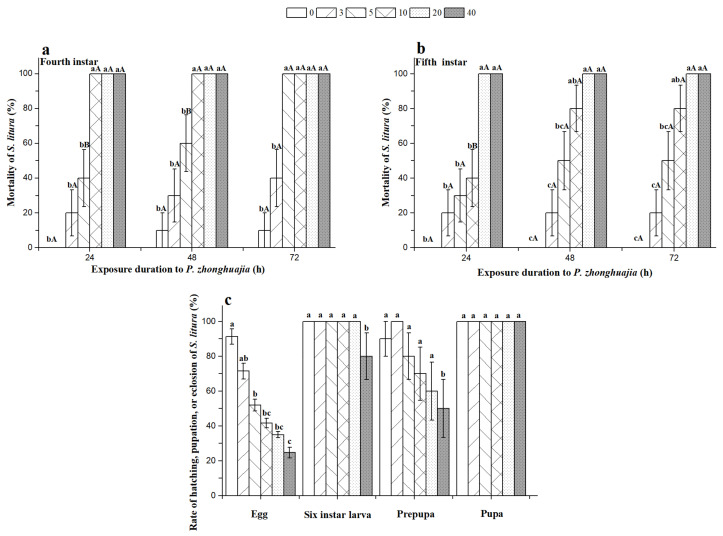
Mortality of fourth to fifth instar larvae. (**a**, **b**) Rate of mortality, hatching, pupation, and eclosion (**c**) of *Spodoptera litura* when exposed to different mite densities (0, 3, 5, 10, 20, or 40 mites). Different lower-case letters on top of bars indicate significant differences in mortality of different densities of *Pyemotes zhonghuajia* to fourth and fifth *S. litura* instar larvae in each exposure duration and in the hatch rate of eggs, pupation of sixth instar larvae, eclosion rate of prepupae, and pupae of *S. litura*. Different capital letters on top of bars indicate significant differences in mortality of fourth or fifth *S. litura* instar larvae at different hours, respectively (Tukey’s honest significant difference (HSD) test, *p* < 0.05).

All test densities of the mites had a significant negative effect on the hatch rate of *S. litura* eggs, and hatch rates reduced with increasing mite population densities (Figure 3). The eclosion rate of pupae were not adversely affected by *P. zhonghuajia*. However, significantly decreased pupation of sixth instar larvae and the eclosion rate of prepupae were observed with 40 mite populations. Mite densities significantly increased the mortality of fourth and fifth instar larvae of *S. litura* (Figure 3). Low densities of mites (three and five mites) did not significantly increase the mortality of fourth and fifth instar larvae of *S. litura*. However, higher densities (20 and 40 mites) significantly increased the mortality of fourth and fifth instar larvae within 24 h.

### 3.5. Searching Ability of P. zhonghuajia on S. litura

**Figure 4:**
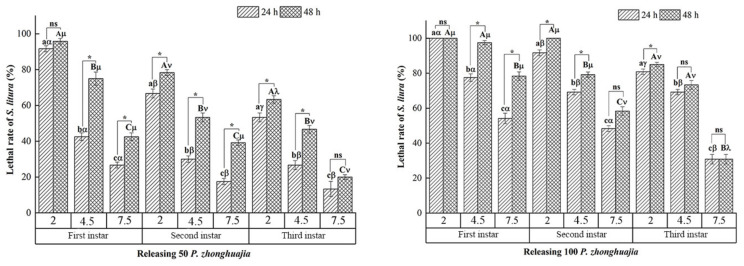
Lethal rates at 24 and 48 h of first, second, and third instar *S. litura* larvae on tobacco leaves under three different searching ranges (with diameters of 2, 4.5, and 7.5 cm) in the presence of 50 or 100 *P. zhonghuajia*. All first, second, and third instar *S. litura* larvae were alive within 48 h in the control without *P. zhonghuajia*. Different lower-case letters (a–c) and capital letters (A–C) on top of bars indicate significant differences in mortality rates of each *S. litura* larval stage between 24 and 48 h, respectively (Tukey’s honest significant difference (HSD) test, *p* < 0.05). The different letters (α, β and γ) and (μ, ν and λ) on top of bars indicate significant differences in mortality rates of *S. litura* between 24 and 48 h. An asterisk (*) indicates that there was a significant difference in the mortality rates of *S. litura* between 24 and 48 h.

When 50 *P. zhonghuajia* were released in three differently sized searching ranges, significantly higher mortality rates of first, second, and third instar *S. litura* larvae were observed in 48 h compared with the mortality rates of *S. litura* larvae in 24 h, except for first instar larvae within a 2 cm range and third instar larvae within a 7.5 cm range. When either 50 or 100 *P. zhonghuajia* were released, significantly higher mortality was generally observed in first, second, and third instar *S. litura* larvae in the smallest searching range (2 cm) than the moth larvae in the 4.5 or 7.5 cm searching ranges at both 24 and 48 h, except for first and third instar larvae at 48 h in the presence of 100 *P. zhonghuajia* (Figure 4). Compared with third instar larvae, the mortality rates of first instar larvae were significantly higher in the presence of 50 or 100 mites in the three searching ranges within both 24 and 48 h.

## 4. Discussion

In this study, we found that individual newly emerged *P. zhonghuajia* were able to paralyze and kill first, second, and third instar *S. litura* larvae. To the best of our knowledge, this is the first study to discover that newly emerging females are capable of killing a third instar larvae greater than 680,000 times its own body weight. *Pyemotes zhonghuajia* mites were able to develop on *S. litura* eggs, larvae, and pupae, but could only produce offspring on eggs and pupae. Mite densities significantly decreased the hatch rate and increased the mortality rate of fourth and fifth instar larvae of *S. litura*, but not sixth instar larvae, prepupae, and pupae. The presence of 50 or 100 *P. zhonghuajia* resulted in significantly higher mortality rates of first, second, and third instar *S. litura* larvae in a 2 cm searching range compared with other searching ranges (4.5 and 7.5 cm). In general, the body weights of various organisms can also change dramatically owing to other factors; for example, the body weight of adult females of the tick species *Haemaphysalis longicornis* can increase by approximately 129.97 times within 6–10 days when engorged with blood compared with hungry ticks [16]. In this study, we first found that in later pregnancy, the weight of *P. zhonghuajia* could increase 14,826 times in only 8 days compared with newly emerging females. To the best of our knowledge, this is the most dramatic reported weight change in any adult female animal during pregnancy in the world. Our results align with previous research showing that host stages can influence the reproductive development of *P. zhonghuajia* [17,18,19]. *P. zhonghuajia* were able to develop normally and produce offspring on the larvae of *Sitotroga cerealella* and *Galleria mellonella*, and the larvae and early pupae of *Sitophilus* spp. [18]. However, *P. zhonghuajia* could only produce offspring on *S. litura* and *M. separata* eggs and pupae, not their larvae [7]. In our study, *S. litura* larvae were shriveled soon after paralysis by *P. zhonghuajia* and could not consistently provide nutrition for the development of these mites. This might be due to the different water retention capacity in the skin of larvae. The larvae of *S. cerealella*, *G. mellonella* and *Sitophilus* spp. are mainly found in relatively dry warehouse environments, while the larvae of *S. litura* and *M. separata* are found in the field (a generally less dry environment) and feed on the leaves of their natural host. The difference in reproductive ability we observed in our study may therefore be due to different natural water retention capacities of larval skin in *S. litura* compared with other hosts, owing to adaptations to different environments. Further research is needed to compare the water retention capacity of larval skin between stored grain pests and agricultural pests parasitized by *P. zhonghuajia*.

*Pyemotes zhonghuajia* could induce rapid muscle contracting paralysis of *S. litura* after infection; similar effects were observed in *P. tritici* on *Ceratomia catalpa*, *Plodia interpunctella*, and *Opisina arenosella* [20,21,22]. The venom of *Pyemotes* spp. is known to contain multiple toxins, including one high molecular weight protein (TxP-HMW) and three low molecular weight proteins (TxP-LMW) [22]. We found that the venom of *P. zhonghuajia* could induce an immediate constriction of *S. litura* first instar larvae within 25 min and cause flaccid paralysis within 1 h. Similarly, Tomalski et al. (1988) found that TxP-LMW causes an immediate muscular constriction of *C. catalpa* within 1 h of injection, and TxP-HMW induces a flaccid paralysis lasting for 4–12 h after injection. Tomalski and colleagues (2006) also reported that the crude toxin extract of *Pyemotes* spp. induced flaccid paralysis in fifth instar *H. armigera* larvae 30 min after injection. Han et al. [11] determined the protein sequences of 12 toxins from *P. zhonghuajia*; the amino acid homology of these toxins and the TxP-LMW sequence in *P. tritici* was over 80%. Lastly, we found that there were two glands in the basal part of the pedipalps in *P. zhonghuajia*; this is consistent with previous research by Weiser and Sláma [10], which described that the venom of *P. zivocljeri* is produced from the glands in the pedipalps. Therefore, we hypothesize that *P. zhonghuajia* may produce low molecular weight venom proteins in the glands of the pedipalps. Future research should therefore focus on the protein components of *P. zhonghuajia* venom and their functional characteristics to better understand the parasitic interactions between this mite and its hosts.

Previous research has shown that single *P. zhonghuajia* females were able to kill single *Yponomeuta* spp. early instar or mature larvae in 27 or 48 min, respectively [23]. This partially aligns with the results of our study, in which single *P. zhonghuajia* females were able to kill single *S. litura* first instar larva in a similar time frame of 37.1 min; however, they were not able to kill fourth, fifth, and sixth instar *S. litura* larvae, prepupae, and pupae within the study period of 720 min. Other studies have also demonstrated a similar lack of killing ability by *P. zhonghuajia* at the same late larval, pupal, and prepupal stages of *S. frugiperda* and *M. separata* within 24 h [6,7].

Previously, mite densities have been shown to significantly influence their lethal efficiency against pests [6,24]. Here, we found that higher densities of mites (20 or 40 per single larva) were able to kill fourth and fifth instar larvae of *S. litura*, but not sixth instar larvae or pupae, within 72 h. The same trend was observed in fourth and fifth instar larvae and prepupae of *S. frugiperda*, and in the fourth instar larvae of *M. separata* [6,7]. In line with previous research, our study also found that parasitism by 20 or 40 *P. zhonghuajia* resulted in up to 50% mortality rates of prepupae of *S. litura*. These mites did not significantly influence the mortality of *S. frugiperda* pupae, but parasitism by mites at these densities can result in up to 100% mortality in *S. frugiperda* prepupae and *M. separata* prepupae and pupae [6,7]. Taken together, this suggests that *S. litura* prepupae and pupae were not particularly susceptible to parasitic killing by *P. zhonghuajia*.

## 5. Conclusions

Our research revealed that the venom of the parasitic mite *P. zhonghuajia* is strongly toxic against *S. litura*, as indicated by our results in this exploration of the paralytic process, time to lethality, lethal efficiency, and searching ability of this mite species in this particular host. To further explore whether *P. zhonghuajia* could be a useful biocontrol agent against *S. litura* and other crop pests, future research should aim to identify, characterize, and understand the functional compounds present in the venom.

## Figures and Tables

**Table 1:** Daily growth and development parameters of *Pyemotes zhonghuajia* on eggs, first to sixth instar larvae, and pupae of *Spodoptera litura*.

Stages	Daily Opisthosomal Growth Equation	a			b		R^2^	Adult Longevity (Days)	Opisthosoma Length (μm)
		Lower 95%CI	Upper 95% CI	Lower 95%CI		Upper 95% CI			
Egg	y = 1028.44(1 − e^(−0.17x)^)	745.05	1311.83	0.08		0.26	0.8	9.6 ± 0.2a	928.33 ± 17.22a
First instar	y = 653.38(1 − e^(−0.2x)^)	341.81	964.94	0.06		0.33	0.77	5.8 ± 0.1d	376.67 ± 29.21d
Second instar	y = 764.36(1 − e^(−0.2x)^)	638.80	889.92	0.15		0.25	0.94	6.6 ± 0.2bc	505 ± 9.64c
Third instar	y = 1010.60(1 − e^(−0.22x)^)	561.60	1459.61	0.10		0.14	0.84	6.7 ± 0.2b	658.33 ± 27.47b
Fourth instar	y =1179.60(1 − e^(−0.12x)^)	366.17	1993.06	0.05		0.25	0.9	6.0 ± 0.0cd	460 ± 61.96cd
Fifth instar	y =1432.49(1 − e^(−0.12x)^)	306.46	2558.52	0.08		0.32	0.84	5.8 ± 0.1d	631.67 ± 77.96b
Sixth instar	y = 959.82(1 − e^(−0.21x)^)	624.17	1295.47	0.09		0.32	0.88	6.8 ± 0.1b	676.67 ± 69.03b
Pupa	y = 1156.99(1 − e^(−0.28x)^)	1083.83	1230.24	0.24		0.32	0.94	9.3 ± 0.2a	971.67 ± 36.05a
F-statistic								F_1,72_ = 105.3	F_1,72_ = 99.774
Significance (*p*)								<0.05	<0.05

The different lower-case letters within a column indicate significant differences in the adult longevity and opisthosoma length (Tukey’s honest significant difference (HSD) test, *p* < 0.05).

## Supplementary Materials

The following are available online at https://www.mdpi.com/article/10.3390/insects12060490/s1, Figure S1: Opisthosoma development of Pyemotes zhonghuajia females on Spodoptera litura eggs. Figure S2: Opisthosoma size of Pyemotes zhonghuajia on different stages of Spodoptera litura. The different lower-case letters in each time near curve indicate that there are significant differences in the opisthosoma size of P. zhonghuajia (Tukey’s honestly significant difference [HSD] test, *p* < 0.05). Figure S3: (a) A ventral view and (b, c) the pedipalps, including the mouthparts and paired venom glands, of an adult female Pyemotes zhonghuajia. Video S1: The parasitic process of *Pyemotes zhonghuajia* on *Spodoptera litura* first instar larvae; Video S2. The parasitic process of *Pyemotes zhonghuajia* on *Spodoptera litura* second instar larvae.
