# Supplementary material for: The Biocontrol Agent Pyemotes zhonghuajia Has the Highest Lethal Weight Ratio Compared with Its Prey and the Most Dramatic Body Weight Change during Pregnancy
Source: Insects. 2021 May 25;12(6):490. doi: 10.3390/insects12060490 (PMC8225142; doi:10.3390/insects12060490)
Supplement: Supplementary file 1 [file insects-12-00490-s001.zip › Supplememtal material.pdf]

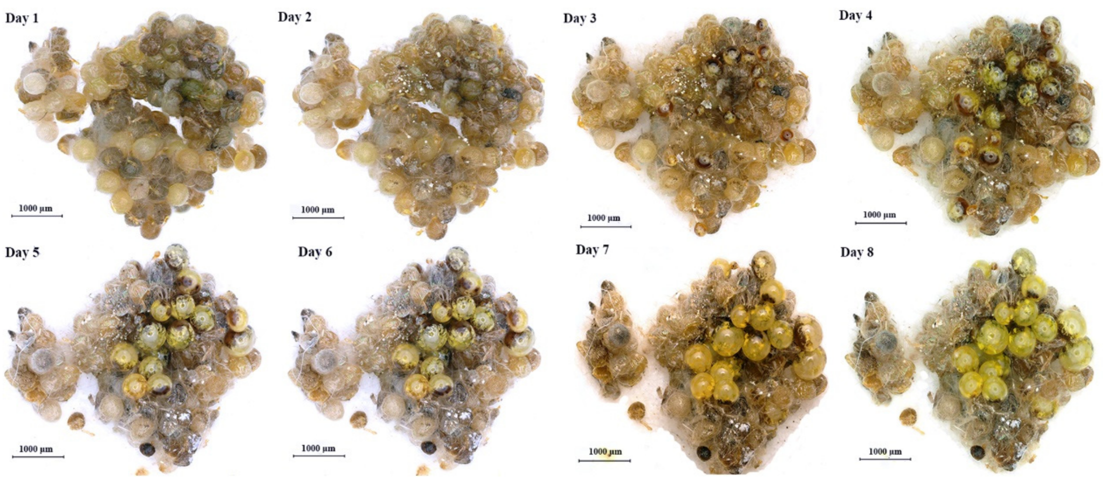

Figure S1. Opisthosoma development of *Pyemotes zhonghuajia* females on *Spodoptera litura* eggs.

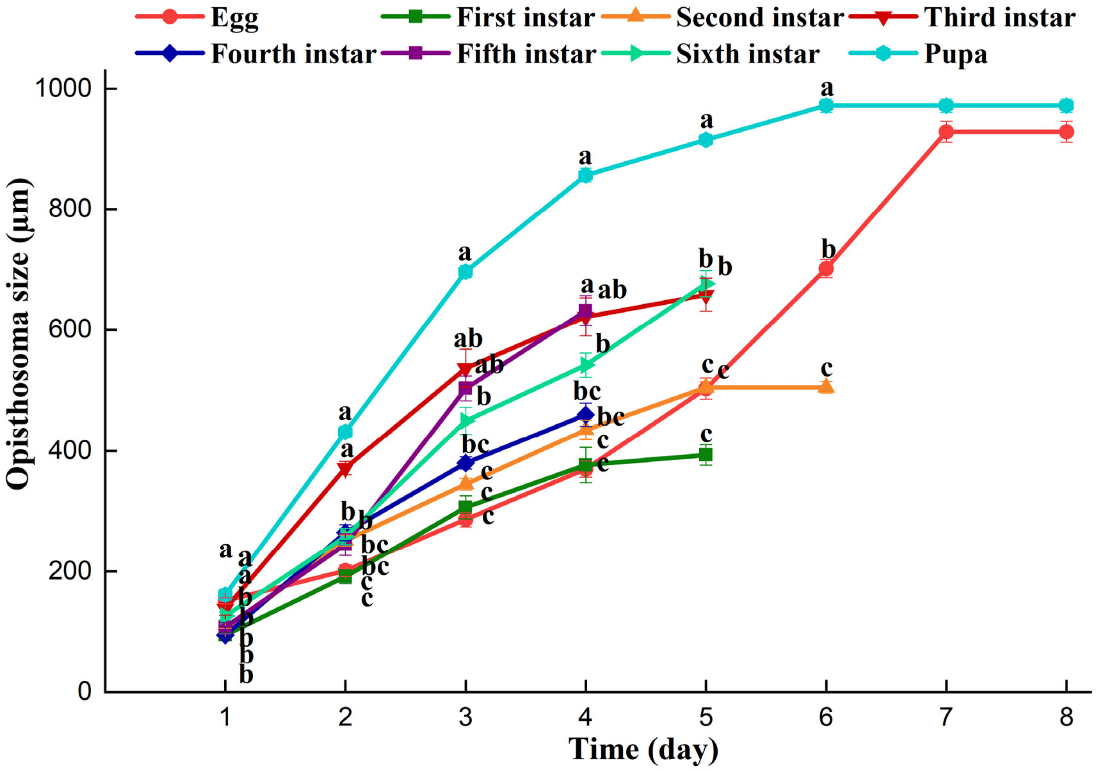

Figure S2. Opisthosoma size of *Pyemotes zhonghuajia* on different stages of *Spodoptera litura*. The different lower-case letters in each time near curve indicate that there are significant differences in the opisthosoma size of *P. zhonghuajia* (Tukey's honestly significant difference [HSD] test, $p < 0.05$).

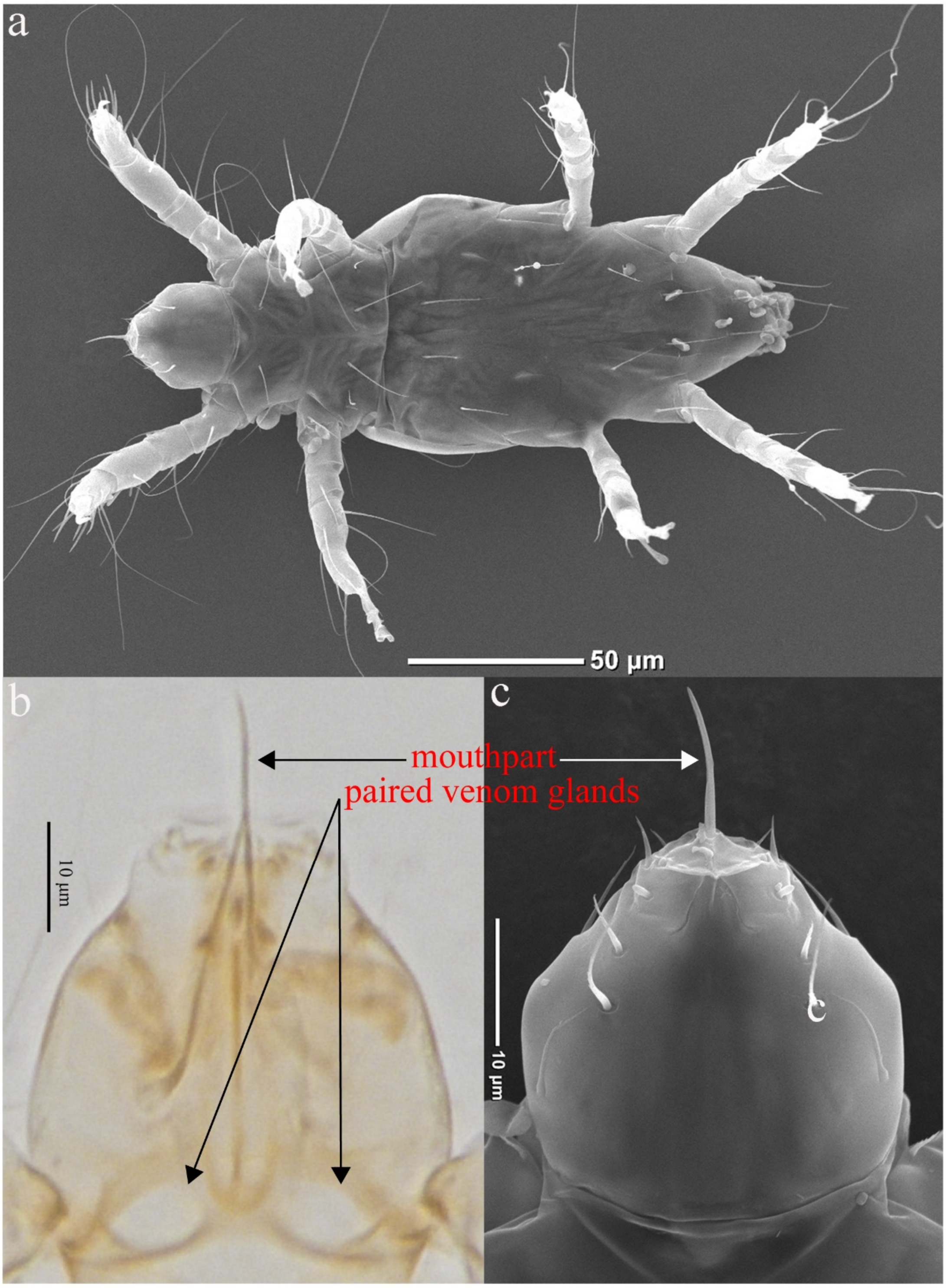

Figure S3. (a) A ventral view and (b, c) the pedipalps, including the mouthparts and paired venom glands, of an adult female *Pyemotes zhonghuajia.*
